# Extracting high-molecular weight DNA from cyanobacteria using Promega's Wizard^Ⓡ^ HMW DNA extraction kit with a modified protocol, METIS

**DOI:** 10.1016/j.mex.2023.102341

**Published:** 2023-08-23

**Authors:** Megan A. Hept, Lesley H. Greene

**Affiliations:** Department of Chemistry and Biochemistry, Old Dominion University, United States

**Keywords:** Aquatic Bacteria, High-molecular weight DNA, Optimization, METIS

## Abstract

Extraction of high molecular weight (HMW) DNA for long read sequencing with little to no fragmentation and high purity is difficult to acquire from cyanobacterial species. Here we describe a modified method of extraction using Promega's Wizard^Ⓡ^ HMW DNA Extraction Kit to acquire high molecular weight DNA from two cyanobacterial species. The protocol used in the kit is the “3.D. Isolating HMW DNA from Gram-Positive and Gram-Negative Bacteria” protocol. During a key step in the protocol, we propose that the lingering remnants of the cellular debris such as the mucilage layer of the cyanobacterial species is removed, preventing it from sticking to the DNA pellet produced. This customized modification is done between steps 11 and 12 and called METIS (maximizing extraction, transfer isopropanol step). This step drastically reduces the remaining mucilage layer, which if kept will stick to the DNA and make the DNA unsuitable for sensitive downstream next generation sequencing, like PacBio Sequencing. This protocol has been used to assemble two genomes from cyanobacteria (*Synechococcus sp.* and *Microcystis aeruginosa*) and one from a gram-negative bacterium, *Lacibacter*. It also allows for HMW DNA to be rapidly extracted without the use of toxic chemicals such as phenol and without extra reagents to be purchased.

• Maximizing extraction, transfer isopropanol step (METIS) is the key modification during the step of DNA unraveling
• METIS reduces leftover remnants of the mucilage layer in the extraction
• High molecular weight DNA is produced with little to no fragmentation, and both a high purity and concentration

## Graphical abstract

**Figure:**
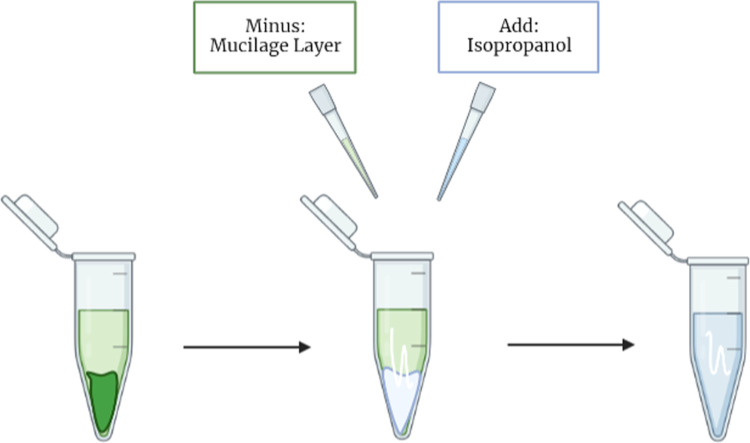

Specifications table

**Table:** 

Subject area:	Biochemistry, Genetics and Molecular Biology
More specific subject area:	*High Molecular Weight DNA Extraction from Cyanobacteria and other Aquatic Bacteria*
Name of your method:	*METIS*
Name and reference of original method:	*Wizard HMW DNA Extraction Kit Technical Manual* *Section: 3.D. Isolating HMW DNA from Gram-Positive and Gram-Negative Bacteria*
Resource availability:	*https://www.promega.com/products/nucleic-acid-extraction/genomic-dna/high-molecular-weight-dna-extraction-kit/?catNum=A2920*

## Method details

Isolating HMW DNA from cyanobacteria for whole genome sequencing is challenging due to the cyanobacteria's unique physiology. Cyanobacteria are gram-negative bacteria that have a cell envelope that shares both gram-negative and gram-positive features and a thick peptidoglycan layer [1]. Surrounding many cyanobacterial cells is a sheath of extracellular polymetric substances (EPS), which is mainly composed of polysaccharides and resembles slime that sticks to the cells and acts as a barrier between the cyanobacteria and its environment [2]. The cyanobacterial physiology, including the mucilage layer that surrounds the cells makes acquiring DNA, especially high molecular weight DNA, a challenge and continued hurdle in cyanobacterial genomics [3].

The protocol presented in this paper is a necessary customization from a commercial kit (Promega Wizard HMW DNA Extraction Kit, Promega Corporation, Madison, WI). In general, kits have an important place in molecular biology as they are by design reliable, rapid, and streamlined procedures that are also far easier to use than purchasing and generating all the reagents individually in the lab. Specifically, we modify the protocol section “3.D. Isolating HMW DNA from Gram-Positive and Gram-Negative Bacteria” [4]. It is not uncommon for researchers to modify methods or generate new methods to produce HMW DNA for sequencing and genetic analysis [5,6]. To the best of our knowledge, there is no known commercial kit with a protocol that is specific for cyanobacteria. Our modified method METIS, short for “maximizing extraction, transfer of isopropanol step”, is designed to drastically reduce the impact contaminants such as the EPS/mucilage layer has on DNA extraction and does so without the use of harsh chemicals, without additional reagents required, and is rapid and cost-efficient. This extra protective layer makes DNA extraction more difficult. There have been past modified methods to address the challenges posed by contaminants [7]. Traditional DNA isolation methods use phenol-chloroform-based DNA extraction procedures, which are also hazardous and therefore less ideal [8]. Refining the DNA extraction technique has led to various enzymatic disruptions to be used, but the EPS layer surrounding the cell offers protection and additional steps for cell lysis are needed [9]. Methods utilizing phenol and lysozyme free protocols have been undertaken [10]. Some methods have explored the combined use of enzymatic and freeze thaw disruption with CTAB precipitation, and have been successful in HMW DNA extraction [11]. A recent study testing several DNA extraction kits (such as Qiagen QIAamp and DNeasy Plant mini-kits and Mo Bio Power Biofilm), were shown to be superior to phenol-chloroform methods [5]. Also, an earlier modified extraction method was successful in acquiring HMW DNA for cyanobacteria but can have some fragmentation [12].

Below are our modifications from Promega's technical manual [1] for the successful extraction of the HMW DNA from cyanobacteria. In summary, in addition to changes in volumes, increases in spin times and incubation periods, the critical customization is the continued use of isopropanol repeatedly to wash the DNA pellet, and successively remove the green debris left over from the mucilage layer. The yield of DNA is dependent on the volume of culture taken for extraction and can be modified as necessary.

1. A key METIS difference is the starting culture amount. While the standard kit protocol recommends 1 mL, we recommend at least 75 mL of a cyanobacterial culture, at its stationary phase, and should be split into two 50 mL tubes.
2. As the METIS modifications call for a larger starting culture, the two 50 mL tubes should be spun down and centrifuge at 16,000 x g for 10 min in a microcentrifuge. The supernatant is removed, and the pellets remain. One pellet should have 1 mL of deionized water added to it, and then mixed. This solution is then added to the other tube and its pellet, mixed, and then relocated to a 2 mL microcentrifuge tube.
3. The standard kit protocol can resume at this stage, and the tube is spun 3 min at 16,000 x g, and the supernatant removed. 100 µl of PBS is added and the pellet is resuspended.
4. The standard protocol calls for the addition of 500 µl of HMW Lysis Buffer A, using a 1,000 µl wide bore tip to mix the solution five times. In this step it is important this is done gently, with a slow draw up and then a rapid, controlled expulsion down the side of the tube.
5. The standard protocol mentions an incubation if the solution is not properly lysed, and this step is necessary for cyanobacteria using the METIS modification. The solution should be incubated at 80 °C for six minutes and then allowed to cool to room temperature. The solution will go from a dark, vibrant green to a dull, pale green after the incubation with heat.
6. Use the standard protocol suggestion of 3 µl of RNase A Solution to the cell solution and invert the tube gently seven times and incubate at 37 °C for 15 min.
7. Use the standard protocol suggestion of 20 µl of Proteinase K Solution to the sample and invert the tube gently ten times and incubate at 56 °C for 15 min. Then chill on ice for 1 min.
8. Use the standard protocol suggestion of 200 µl of Protein Precipitation Solution to the solution, and then mix gently five times using wide bore a 1,000 µl pipette tip. A slow draw up and then a rapid, controlled expulsion down the side. The tube is then incubated on ice for 5 min.

Tips:

• The protocol says if there are no wide bore tips to vortex. Do not vortex, cut the regular tips to resemble wide bore.

9. A key METIS difference is that the solution will then need a longer centrifuge step than the standard protocol and depending on the amount of culture used, will potentially need extra time as well. Centrifuge the tube at 16,000 x g for 15 min to begin. If there are any wisps of darker green (parts of the mucilage layer) that are not solidly packed down in the protein pellet, respin for 5 more minutes or longer as necessary. The tighter the protein pellet the easier the extraction in the next step will be.

Tips:

• The protein pellet should be a dark green, while the supernatant should be a light green with no visible clumps or wisps seen. Color of the supernatant will vary depending on the amount of culture used.

The next steps are key to the METIS technique, and it is good to have on hand the following before starting this stage. This will take time and can take up to 30 min to do per tube.

○ 10 mL of isopropanol
○ 200 µl regular tips
○ Two 200 µl pipettes, one to take out the supernatant, the other to be used to add isopropanol

10. The standard protocol is used first, by uptaking the supernatant in the tube with the protein pellet using a wide bore 1,000 µl pipette tip and then adding this supernatant to a new tube that contains 600 µl of isopropanol.

Tips:

• Try to avoid pipetting up any of the mucilage layer that has been packed down into the protein pellet. It is better to leave a little supernatant in the tube then risk adding any of the mucilage layer into the tube with the isopropanol and the unraveling DNA.
• A key METIS difference: the Promega protocol calls for the supernatant to be poured into the fresh tube. If you do this, there is a high chance the mucilage layer that has been spun down into the protein pellet will get dislodged and pour into the tube as well. Use a pipette to move supernatant.

11. Using the standard kit protocol, the tube will now have the green supernatant and the isopropanol, and needs to be gently inverted 8 times and incubated for 1 min. A pellet of DNA will start to unravel and be visible. This is a key stage and care must be taken to be gentle.
12. In the tube, there is now a green supernatant, and the objective of METIS is to remove the green debris and leave the DNA in a tube of pure isopropanol. A key METIS modification is how to extract this lingering green supernatant. To do this, the green solution must be extracted carefully, and then replaced with the isopropanol. Using a 200 µl tip, carefully withdraw up to 200 µl of supernatant. Replace with 200 µl of isopropanol. Repeat until the solution is clear and there is little to no mucilage layer leftover.

Tips:

• It is easier to do this step if the DNA is clumped together and not in a string like form. If the DNA is not clumped together, slowly inverting the tube sideways and rock gently from one side to the other. When taking out the supernatant you must be careful not to pipet in the DNA pellet. Err on the side of caution and only uptake small aliquots at a time to avoid this. If the DNA pellet is drawn in or stuck at the tip opening, you risk the DNA being sheared or broken.
• When adding the new isopropanol to the tube and supernatant add it gently to the side of the tube. Do not forcefully expel isopropanol into the solution. This could break the DNA or make the remaining mucilage layer stick to the pellet.
• The key to this is patience and time, do not rush this step and be careful regarding the DNA pellet.

13. After a visible DNA pellet is observed in clear supernatant, the regular standard kit protocol can resume. The tube is then centrifuged for 2 min at 16,000 x g, and the DNA pellet should be visible.
14. Using the standard kit protocol, pour out the supernatant and add 600 µl of 70% ethanol to the DNA. Gently invert the tube 7 times to wash the DNA pellet and then centrifuge the pellet again for 2 min at 16,000 x g.
15. Using the standard kit protocol, decant the supernatant with care to avoid losing the DNA pellet. The tube is then inverted on clean absorbent paper and allowed to air dry for 15 min.
16. Using the standard kit protocol, add 200 µl of DNA Rehydration Solution to the tube and then incubate for 1 h at 65 °C. Every 15 min mix the solution by gently tapping on the sides.
17. If the pellet is dissolved, take the DNA solution and then mix gently with a wide bore 200 µl tip until homogeneous. A METIS difference is to do an additional wait step and store this DNA for 24 h at 4 °C before attempting to quantify it, concentrate it, or run it on an agarose gel. This should allow maximum homogeneity to occur before downstream work is performed.

## Method Validation

Validation of the METIS modification to the Promega protocol was tested by using two difference species of cyanobacteria and performing extractions in a number of replications. The extracted DNA was visualized using agarose gels and PacBio sequencing performed to show proof of ability to acquire long read sequencing from cyanobacteria using this modified method.

**Fig. 1:**
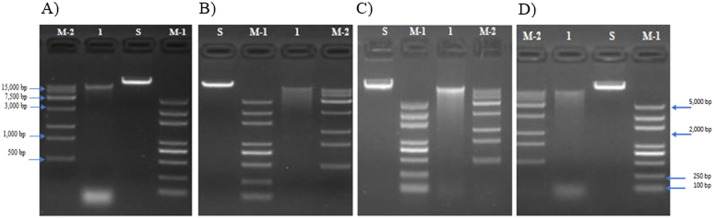
Gel image of DNA extracts. (A–D) *Synechococcus sp.* DNA extracted using the standard protocol from the Promega Wizard HMW Kit. Molecular marker ladders are shown in lanes M1 and M2. A standard (S) was run as a control for HMW DNA. Samples are run in lanes marked 1. Gels were 1% agarose and run on 180 V for 20 min. Images were taken by the company Novogene.

**Fig. 2:**
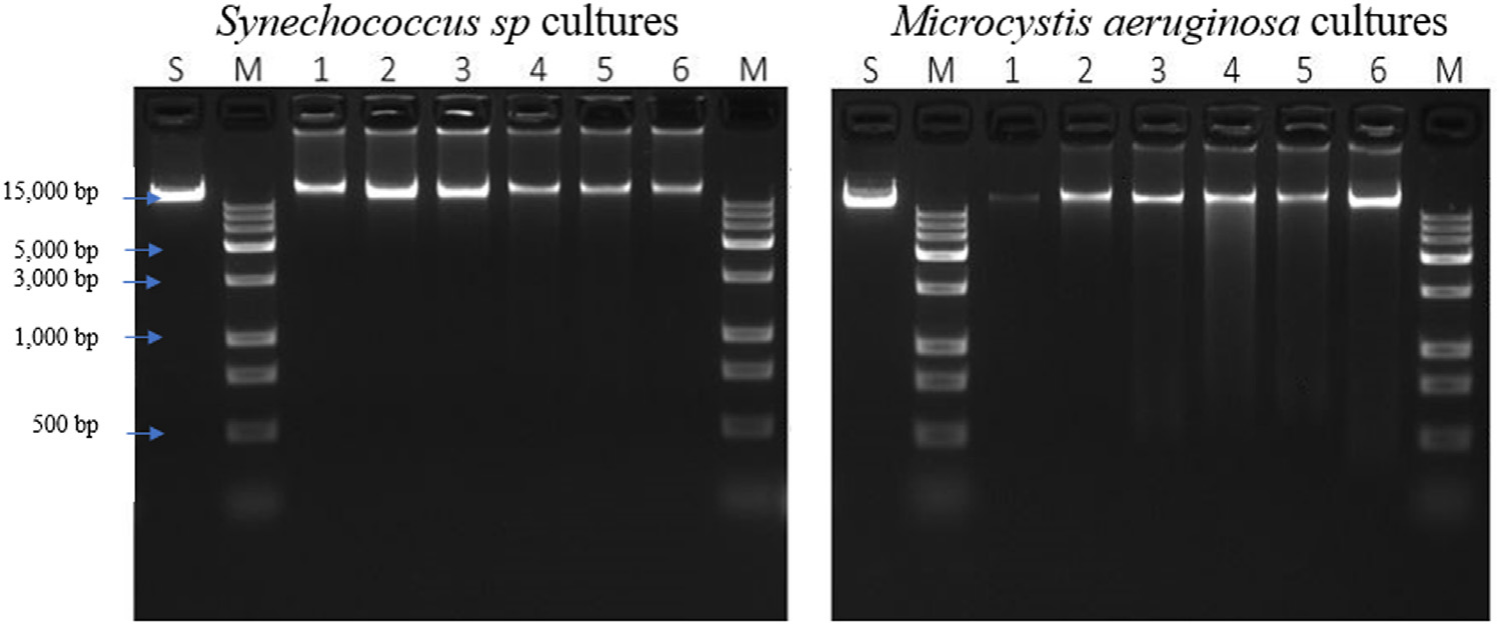
Gel image of HMW DNA extracted with the METIS method for two different cyanobacterial cultures. A standard control HMW DNA was used for comparison and is denoted with an S. Molecular marker is denoted with M. Sample cultures are denoted with the numbers 1–6. Gels were 1% agarose and run on 100 V for 40 min. Gel images produced by the company Novogene.

**Fig. 3:**
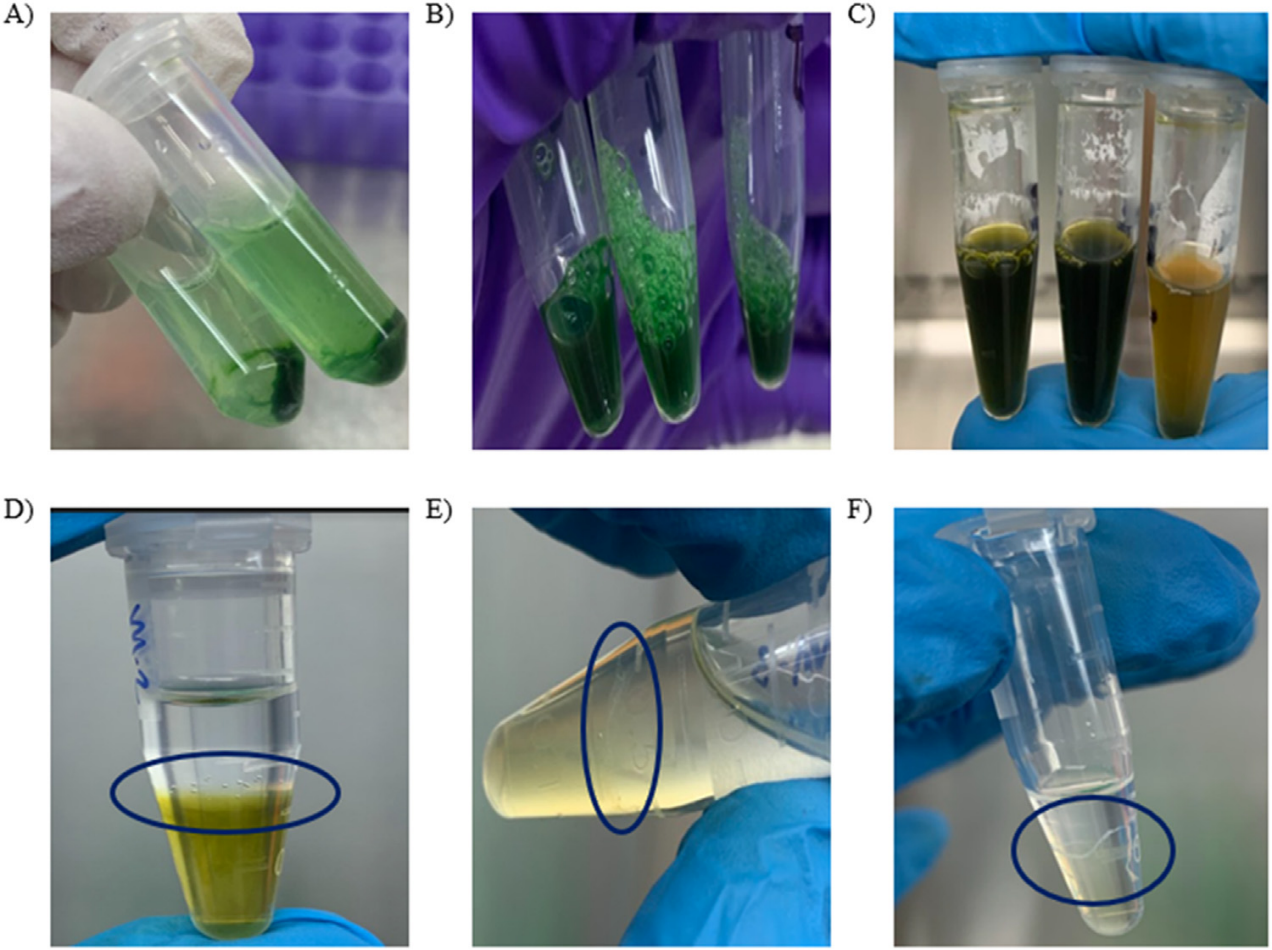
Key steps and problems in the extraction process and a visual representation as guidance during the METIS extraction. (A) Step 2: The pellet and the mucilage layer that surrounds it requires that care must be taken when removing the supernatant to not disturb this. (B) Step 4: Addition of the HMW lysis buffer after mixing but is not lysed as evident by the dark green of the solution. (C) Step 5: After heating at 80 °C for six minutes, a color change should be seen. In (D–F) the dark blue circle indicates where the DNA is unraveling or already exposed. (D) Step 11: The isopropanol and the cellular debris such as the mucilage layer are separated, and the cluster of bubbles shows where the DNA is being unraveled. (E) Step 12: Example of the METIS process with the contaminants slowly being replaced with fresh isopropanol. (F) Step 12: Example of visible, cleaner DNA using the METIS process.

When using the Promega Wizard HMW kit with the protocol for gram-positive and negative bacterium without the METIS method, DNA is produced that is fragmented and smeared (Fig. 1). The METIS modification was tested on two different types of cultures, one pure culture of a *Synechococcus sp*. and one mixed culture of *Microcystis aeruginosa* LE3 and *Lacibacter sp*. The HMW DNA extracted was then run on an agarose gel and showed several key characteristics: a major single band with little to no degradation, a band that is over 15,000 base pairs in length, and a bold band indicates a higher concentration (Fig. 2). Using the METIS modifications, the DNA extracted has a consistent higher purity than DNA extracted using the standard protocol (Table 1). The HMW DNA taken from these cultures was shipped to the biotech company Novogene for PacBio sequencing by a DNA CLR library using a Pacific Biosciences Sequel II platform. Illumina reads were sequenced from the HMW DNA extracted from the METIS method for the mixed culture of *M. aeruginosa* and a species of *Lacibacter* and produced using a pair-read library on an Illumina NovaSeq 6000 (Table 2). Key steps of the protocol and a visual representation of some of the key stages of the extraction are shown in (Fig. 3).

**Table 1:** Comparison of purity yields using the standard protocol and the METIS modifications.

Extraction method	260/280 Range
Promega 3.D protocol	1.4–1.9
Promega 3.D protocol with METIS modifications	1.8–2.0

**Table 2:** Raw reads produced by using the METIS method.

Organisms	Sequencing performed	Number of Reads Produced
*Synechococcus sp.*	PacBio	416,285
*Microcystis aeruginosa/Lacibacter sp.*	PacBio	82,609
*Microcystis aeruginosa/Lacibacter sp.*	Illumina	14,124,202

## Ethics statements

No humans or animals were used for this work.

## Funding

This work was supported in part by a Small Project Research Grant from the Virginia Academy of Sciences.

## CRediT authorship contribution statement

**Megan A. Hept:** Conceptualization, Methodology, Data curation, Visualization, Validation, Writing – original draft. **Lesley H. Greene:** Supervision, Writing – review & editing.

## Declaration of Competing Interest

The authors declare that they have no known competing financial interests or personal relationships that could have appeared to influence the work reported in this paper.

## Data Availability

Data will be made available on request.
